# Supplementary material for: Effects of individual and organizational factors on job tenure of primary care physicians: A multilevel analysis from Brazil
Source: PLoS One. 2023 Apr 27;18(4):e0271655. doi: 10.1371/journal.pone.0271655 (PMC10138250; doi:10.1371/journal.pone.0271655)
Supplement: Attachment — Submitted filename: PONE-D-22-18937_KH.pdf [file pone.0271655.s001.pdf]

The effect of individual and contextual factors on the high turnover of primary care physicians: Findings from a multilevel multivariate analysis of a representative cohort in Sao Paulo, Brazil

Comments to the Author(s)

Purpose of the study

This study aims to investigate the job tenure among physicians in primary care (n = 2,335). It seems difficult to me to sum up the contents, because many aspects remain unclear to me, above all, because concepts or variables are named differently throughout the manuscript.

Major comments

1. Whole manuscript: According to the abstract, results show that job tenure is associated with age and professional experience as well as being specialised in primary health care. These results do not seem very relevant to me. Is it not logical that people working longer in a certain position tend to be older and more experienced – simply because they spent more time on their job?

   However, in the "Results"-section, the wording is different: "age at the time of hire" (lines 189f.) and "time elapsed since the completion of medical school and hire in a PHCU" (lines 196f.) are identified as protective factors against turnover, amongst others.
    a) The two factors seem to be intertwined – people who completed school longer ago may tend to be older when beginning a new job. How did you account for the potential interdependence of the predictors in your statistical model?
    b) Please use terms consistently throughout your manuscript. Please make sure that the information in your abstract corresponds to the information in your manuscript

2. Whole manuscript: My major concern – beside the fact that the results do not seem very relevant to me – is that several concepts are mixed in this manuscript. In the title, "turnover" in mentioned. The abstract mainly refers to "tenure". Only in the conclusion, "turnover" is brought up again.

   On the one hand, the outcome of this study is defined the "length of time from the date of hire to the termination of the physician's employment in PHC services" (line 96). This sounds like "job tenure" (and a continuous variable) to me. On the other hand, in the results, protective factors against termination are identified, regardless of the duration, i.e., the job tenure. This sounds like "turnover" (and a dichotomous variable) to me.

   Therefore, my suggestion is to adjust title, abstract, and the whole text (maybe even statistical analyses) completely to the concept of "job tenure" OR to the concept of "turnover" including a definition in the introduction and background.

3. Introduction: Please extend the introduction and background. In this section, you might want to present prior evidence on factors that are associated with job tenure/retention/intention to stay OR turnover behaviour among health care professionals. Before this background, you might want to state more explicitly how your research contributes to the field.

4. Please state a concrete study aim in your manuscript.

Minor comments

5. Section “Variables, outcome definitions and ethical considerations”: In the abstract, you explain: “Human resource data from two distinct databases were used.”
    a) As I could not find this information in the methods-section, please insert it there.
    b) Please describe who collected the data for the two databases, respectively.
    c) Please give more specific information on the variables, e.g., gender: Was there a third option?, What kinds of specialisations were given – or was it an open text field? How was professional experience operationalised?, How was workload operationalised?
    d) Please specify if you did some recoding of the variables (e.g., dichotomisation) – and if so, how you did that.

6. Page 5, lines 108-110: Please state the number of the Ethics Committee approval.
7. Page 5, line 114: Please give names of enterprises distributing the software you used.
8. Page 6, line 140: Please explain the term “person-time”.
9. Page 6, line 141: Please revise number “26,113.741 months”.
10. Page 6, lines 141f.: If the outcome of this study is the “length of time from the date of hire to the termination of the physician’s employment in PHC services”, how can participants still be “actively employed in PHC”? Please explain.
11. Table 2: Please explain all abbreviations used in the table in a note below.
12. Table 2: As far as I understand it, “workload” and “weekly working hours” are different concepts.
    a) Please change the name of your variable or give a rationale why you operationalise “workload” as “weekly working hours”.
    b) Please state if the weekly working hours are the hours specified in the contract or the actual working hours including overtime.
13. In the title, you state that the data stem from a “representative cohort” – please explain how you ensured or checked that your sample is a representative one for all physicians in primary care in your study region.
14. Page 7, lines 149f.: Please name and define all your variables in the “Methods”-section.
15. You might want to start the discussion with a short summary of all your results.
16. Page 10, line 243: You may want to revise the beginning of the sentence.
17. Page 13, lines 308f.: “For instance, because the excluded physicians were hired by organizations other than the three employers studied …” I did not quite catch that fact before. Please explain in your methods why you focused on three employers only.
