# Supplementary material for: Effects of individual and organizational factors on job tenure of primary care physicians: A multilevel analysis from Brazil
Source: PLoS One. 2023 Apr 27;18(4):e0271655. doi: 10.1371/journal.pone.0271655 (PMC10138250; doi:10.1371/journal.pone.0271655)
Supplement: Attachment — Submitted filename: Response to Reviewers.docx [file pone.0271655.s002.docx]

December 10, 2022

**Revision of manuscript PONE-D-22-18937**

**The effect of individual and contextual factors on the high turnover of primary care physicians: Findings from a multilevel multivariate analysis of a representative cohort in Sao Paulo, Brazil**

Dear Prof. Adrian Loerbroks

Academic Editor, Plos One

Thank you for the opportunity to submit a revision of our manuscript to the Plos One, and for the insightful comments received*.*

This document includes all of our responses to reviewers' comments.

The study includes anonymous information of physicians who worked in primary care services administered by third party organizations. Restriction to sharing de dataset was imposed by the Research Ethical Committee of the Medical Faculty of São Paulo University. Data from this paper are available upon request to the Ethics Committee of the medical School of the University of São Paulo. Mailing address: 251 Dr. Arnaldo Avenue- Cerqueira César – 01246-000 – São Paulo – SP – Brazil. Phone: + 55 (11) 3893–4401. E-mail: [cappesq.adm@hc.fm.usp.br](mailto:cappesq.adm@hc.fm.usp.br)

Regarding the image presented in Figure 1, the map comes from an open-source public institute (Brazilian Institute of Geography and Statistics - IBGE) that specifically allows reproduction to publish these figures specifically under the CC BY 4.0 license. Please note IBGE background maps have been used by researchers in Brazil for the last 30 years with no property rights restriction.

Please do not hesitate to contact me if you have further comments or questions.

Sincerely,

**Ivan Wilson Hossni Dias**

***Corresponding author***

**Editors’ comments**

1. *Please ensure that your manuscript meets PLOS ONE's style requirements, including	those for file naming.*

Our response: We change file naming, including the separate file labeled 'Revised Manuscript with Track Changes' and the revised paper without tracked changes as a separate file labeled 'Manuscript'. We adjusted the headings with Level 1 18pt font.

2. *Please state what role the funders took in the study. If the funders had no role, please state: "The funders had no role in study design, data collection and analysis, decision to publish, or preparation of the manuscript." If this statement is not correct you must amend it as needed. Please include this amended Role of Funder statement in your cover letter; we will change the online submission form on your behalf.*

Our response: We included in Funding Statement that "The funders had no role in study design, data collection and analysis, decision to publish, or preparation of the manuscript." (Lines 400-402)

3. *We note that you have indicated that data from this study are available upon request. PLOS only allows data to be available upon request if there are legal or ethical restrictions on sharing data publicly. For more information on unacceptable data access restrictions, please see http://journals.plos.org/plosone/s/data-availability#loc-unacceptable-data-access-restrictions. In your revised cover letter, please address the following prompts:*

*a) If there are ethical or legal restrictions on sharing a de-identified data set, please explain them in detail (e.g., data contain potentially sensitive information, data are owned by a third-party organization, etc.) and who has imposed them (e.g., an ethics committee). Please also provide contact information for a data access committee, ethics committee, or other institutional body to which data requests may be sent.*

*b) If there are no restrictions, please upload the minimal anonymized data set necessary to replicate your study findings as either Supporting Information files or to a stable, public repository and provide us with the relevant URLs, DOIs, or accession numbers. For a list of acceptable repositories, please see http://journals.plos.org/plosone/s/data-availability#loc-recommended-repositories.*

*We will update your Data Availability statement on your behalf to reflect the information you provide.*

Our response: The study includes anonymous information of physicians who worked in	primary care	services administered by third party organizations. Restriction to sharing	the dataset was imposed by the Research Ethical Committee of the Medical	Faculty of São Paulo	University. Anonymized data from this paper would be available	upon request to the	Ethics Committee of	the medical School of the University of São	Paulo. Mailing	address: 251 Dr. Arnaldo	Avenue- Cerqueira César – 01246-	000 – São Paulo – SP	– Brazil. Phone: + 55 (11)	3893–4401

4. *Please ensure that you include a title page within your main document. You should list all authors and all affiliations as per our author instructions and clearly indicate the corresponding author.*

Our response: We included a title page with all authors and their affiliations listed, and	actualized information of the third author (Giuliano Russo, Wolfson Institute of	Population Health, Queen Mary University of London, Email: <[g.russo@qmul.ac.uk](mailto:g.russo@qmul.ac.uk)>,	ORCID: 0000-0002-2716-369X)

5. *Your ethics statement should only appear in the Methods section of your manuscript. If your ethics statement is written in any section besides the Methods, please move it to the Methods section and delete it from any other section. Please ensure that your ethics statement is included in your manuscript, as the ethics statement entered into the online submission form will not be published alongside your manuscript.*

Our response: We included the ethics statement in Methods section (page 7, 3^rd^ paragraph, lines170-180) and deleted from the Ethics Section.

6. *We note that Figure 1 in your submission contain [map/satellite] images which may be copyrighted. All PLOS content is published under the Creative Commons Attribution License (CC BY 4.0), which means that the manuscript, images, and Supporting Information files will be freely available online, and any third party is permitted to access, download, copy, distribute, and use these materials in any way, even commercially, with proper attribution. For these reasons, we cannot publish previously copyrighted maps or satellite images created using proprietary data, such as Google software (Google Maps, Street View, and Earth). For more information, see our copyright guidelines:* [*http://journals.plos.org/plosone/s/licenses-and-copyright*](http://journals.plos.org/plosone/s/licenses-and-copyright)*.*

*a. You may seek permission from the original copyright holder of Figure 1 to publish the content specifically under the CC BY 4.0 license. We recommend that you contact the original copyright holder with the Content Permission Form (http://journals.plos.org/plosone/s/file?id=7c09/content-permission-form.pdf) and the following text:*

*“I request permission for the open-access journal PLOS ONE to publish XXX under the Creative Commons Attribution License (CCAL) CC BY 4.0 (http://creativecommons.org/licenses/by/4.0/). Please be aware that this license allows unrestricted use and distribution, even commercially, by third parties. Please reply and provide explicit written permission to publish XXX under a CC BY license and complete the attached form.”*

*Please upload the completed Content Permission Form or other proof of granted permissions as an "Other" file with your submission.*

*In the figure caption of the copyrighted figure, please include the following text:	“Reprinted from [ref] under a CC BY license, with permission from [name of publisher],	original copyright [original copyright year].”*

*b. If you are unable to obtain permission from the original copyright holder to publish	these figures under the CC BY 4.0 license or if the copyright holder’s requirements are	incompatible with the CC BY 4.0 license, please either i) remove the figure or ii) supply	a replacement figure that complies with the CC BY 4.0 license. Please check copyright	information on all replacement figures and update the figure caption with source	information. If applicable, please specify in the figure caption text when a figure is	similar but not identical to the original image and is therefore for illustrative purposes	only.*

Our response: The map presented in Figure 1 comes from an open-source	public institute (Brazilian Institute of Geography and Statistics - IBGE) that specifically	allows reproduction to publish these figures specifically under the CC BY 4.0 license.	IBEGE maps are not subject to any copyright protection and have been extensively	used to conduct research in Brazil for the last 30 years – see IBEGE policy on maps	sharing here < https://www.ibge.gov.br/en/geosciences/maps/state-maps.html>.

7. Please note that funding information should not appear in the Acknowledgments section or other areas of your manuscript. We will only publish funding information present in the Funding Statement section of the online submission form. Please remove any funding-related text from the manuscript (edit requested December 8^th^ 2022).

Our response: We removed the funding information from the manuscript (page 16, Funding-text removed).

8. We note that your Data Availability Statement reads: "The study includes anonymous information of physicians who worked in primary care services administered by third party organizations. Restriction to sharing de dataset was imposed by the Research Ethical Committee of the Medical Faculty of São Paulo University. Data from this paper are available upon request to the Ethics Committee of the medical School of the University of São Paulo. Mailing address: 251 Dr. Arnaldo Avenue- Cerqueira César – 01246-000 – São Paulo – SP – Brazil. Phone: + 55 (11) 3893–4401"

   At this time, please provide direct, non-author email contact information for the body imposing the restrictions upon the data, to which data access requests can be sent. This information will be helpful in updating your Data Availability Statement (edit requested December 8^th^ 2022).

Our response: We included the e-mail for the Ethics Committee (page 16, line 399) to which data access requests can be sent.

**Reviewer #1**

*Dear authors,*

*I was a pleasure reading your manuscript.*

*It is generally well written with a clear structure, well applied research methods and appropriate conclusions. The topic is very relevant. The language is easy to read in proper English. I found no typos.*

Our response: We thank the reviewer’s positive comments and contributions that improve the quality of the manuscript.

*1. Abstract: Please provide no unclear abbreviations here (PHCUs, PHC …), which have not been introduced before*.

Our response: The abbreviation of the Primary Health Care Units was excluded from the abstract. Although, we maintain abbreviation of Primary Health care because of its traditional use in family medicine and public health.

*2. Introduction: It would help, if you could describe in a few sentences how primary health care is organized in Brazil. E.g. in Germany < 1% of these physicians are hired by private companies. Furthermore, once in a practice these physicians usually stay there for their whole working life. I guess the GP system in the English NHS is quite different as well with a far lower turnover.*

Our response: We thank the reviewer for the important comment. We included a new paragraph (page 4, second paragraph, lines 83-90) that briefly describes the primary care organization in the setting of the study.

*3. What are the physicians doing after quitting their PHC jobs?
4. Page 8, line 174: Do you know, if the ones who worked parttime often had children/ family to take care of?*

Our response: We thank the reviewer for the important comment. We cannot examine the marital status of the physicians and what are they doing after quitting their PHC jobs due to limitations of the database.

**Reviewer #2**

*Globally, there is an increasing worry about health workforce shortages in healthcare settings with LMICs experiencing the severest form. The capacity of the health workforce in these regions is insufficient to meet the population’s health objectives; and this disparity is an important limitation in realizing the health-related SDGs. With it potential financial costs and adverse effect on quality patient care, physician turnover is an important subject of interest. The authors are therefore commended for researching into the contextual issues that influence turnover of physicians.*

Our response: We thank the reviewer’s positive comments and contributions to improve the quality of the manuscript.

***Title*** *1. The title can be made concise: “Effect of individual and organizational factor on turnover of PC physicians: A multilevel analysis in Brazil”*

Our response: We accept the suggestion for the title to **“Effects of individual and organizational factors on turnover of primary care physicians: A multilevel analysis from Brazil”**

***Abstract*** *2. What about middle income countries such as Brazil (page 1 line 7); consider including the happening in middle-income countries too*

Our response: We included the happening in middle-income countries (page 2, line 30).

*3. The objective of the study was well stated*

Our response: We thank the reviewer comment.

*4. What’s the significance of reporting the median? Any reason? Page 1 line 20. I recommended using the mean if the assumptions have not been breached*

Our response: We prefer to use the median due to the asymmetry of the distribution of the time of physician tenure in the Primary Health Care Units which is right skewed.

***Keywords***

*5. Arrange keywords alphabetically*

Our response: We rearranged keywords alphabetically (lines 54-55).

***Introduction and background***

*6. Reconcile the use of the term medium (Middle)-income countries page 3 line 38*

Our response: We corrected the term medium to middle-income countries (page 3, line 59).

*7. Provide reference for page 3 lines 38-41*

Our response: We included the reference to the statement in 1^st^ paragraph, lines 59-62.

*8. The import should not be hierarchical analysis but factor that contribute to turnover of physicians*

*9. Contextual factors as used in the title can be explain in detail in the background than what has been done.*

*10. I recommend adding hypothesis as the authors used regression analysis among variables*

Our response (issues 8,9,10): We included a new paragraph to address these suggestions (page 4, 2nd paragraph) including all new description of contextual factors and add hypothesis with respect to the individual and contextual variables considered in the background of the study as follows:

“In the last decade, Brazil has experienced an increase in newly graduate doctors, the majority from private medical schools. Desire for immediate financial gains and the difficulty in accessing specialty training are some aspects that could make these professionals seek PHC services with reduced tenure [19]. Regarding the organizational characteristics, primary care physicians are employed directly by the State or third-party organizations, named Social Organizations [19]. Differences between recruitment politics, selection and incentives applied by these employing organizations are contextual factors that may affect tenure of the physicians in PHC.”

***Method***

***Study design, research scenario and inclusion criteria***

*11. Was the study a retrospective study or retrospective cohort study? Page 3 line 70. Reconsider it. What makes it a cohort study?*

Our response: This is a retrospective cohort study without control group. Primary care physician employed by the biggest three Social Organizations in Sao Paulo city working in Primary Health Care Units were accompanied from January 1, 2016, to July 17, 2020. According to epidemiological textbook (Rothman KJ, Greenland S, Lash TL. Modern Epidemiology. 3rd ed. Philadelphia: Lippincott Williams & Wilkins; 2008) this can be considered a cohort study because individuals (primary care physicians) with a common exposure (work in Primary Health Care Units) were followed over time, starting form a well-described inception point.

*12. Did the study consider the data of only physicians who have terminated their contract or those in employment were also considered? Page 4 line 77 to 80. If both categories were considered, why retrospective study? Is it not advisable to consider those who have terminated their employment as actual and those in employment as perceived? The write up in page 4 line 96 to 99 do not match the earlier assertion as the analysis concentrated on physicians who terminated their contract in the period under review*

Our response: The tenure was calculated from the difference between the date of initial employment and the date of the physician’s termination of contract from PHC up until the date of the closing of the database in December 31st, 2016. We have adjusted the 2nd paragraph, page 6 (lines 131-136) to clarify the outcome and the state variable of survival analysis.

***Data analysis***

*13. At what significance was the analysis done?*

*14. Page 5 line 124; a significance criterion of 0.20 was applied; any justification for the value?*

Our response (issues 13,14): We applied a significance criterion of 0.2 as considered in the reference [26] (Chowdhury MZI, Turin TC. Fam Med Com Health 2020;8:e000262. doi:10.1136/fmch-2019-000262). The authors recommend using a p-value range 0.15-0.20 to guarantee that important variables that may have practical reasoning are not missed.

***Results***

*15. I recommend that the authors present only the key findings by summarizing the descriptive results from page 6 line 135 to page 8 line 180*

Our response: We remake the Results, excluding first paragraph (page 8), summarized demographic variables and professional experience in 2^nd^ paragraph (lines 187-191) and aspects of medical education program (page 9, 1^st^ paragraph, lines 199-203).

***Discussion***

*16. Did the study consider the variables stated in page 12 line 290 to 293? I recommend the authors stick to the objective of the study*

Our response: We adjusted this paragraph with respect to the study objectives, as modified in page 14, 1^st^ paragraph lines 325-326.

*17. Any implications to report for healthcare mangers? Also indicate potential areas for future research*

Our response: As pointed in 4th paragraph lines 362-371, our study shows that physician turnover in PHC is closely related to the physician’s career, explaining why contract variables such as ‘salary’ and ‘weekly workload’ do not play a significant role in the tenure of physicians in Primary Health Care Units. Organizational factor, including differences between employing organizations and Primary Health Care Units are relevant and reveals the importance of investments in service infrastructure of Primary Health Care Units that could imply in an increase of tenure in PHC.

***References***

*18. References well written, I will however recommend that the doi is added to the reference list*

Our response: We included all doi to the reference list.

**Reviewer #3**

*The effect of individual and contextual factors on the high turnover of primary care physicians: Findings from a multilevel multivariate analysis of a representative cohort in Sao Paulo, Brazil*

***Purpose of the study***

*This study aims to investigate the job tenure among physicians in primary care (n = 2,335). It seems difficult to me to sum up the contents, because many aspects remain unclear to me, above all, because concepts or variables are named differently throughout the manuscript.*

Our response: It is sad the reviewer struggled to identify the key contents in our work. We have now tried to streamline the manuscript to make the key messages more prominent and improve the fluidity of the arguments.

***Major comments***

*1. Whole manuscript: According to the abstract, results show that job tenure is associated with age and professional experience as well as being specialised in primary health care. These results do not seem very relevant to me. Is it not logical that people working longer in a certain position tend to be older and more experienced – simply because they spent more time on their job?*

*However, in the “Results”-section, the wording is different: “age at the time of hire” (lines 189f.) and “time elapsed since the completion of medical school and hire in a PHCU” (lines 196f.) are identified as protective factors against turnover, amongst others.
a) The two factors seem to be intertwined – people who completed school longer ago may tend to be older when beginning a new job. How did you account for the potential interdependence of the predictors in your statistical model?*

*b) Please use terms consistently throughout your manuscript. Please make sure that the information in your abstract corresponds to the information in your manuscript*

Our response: Our study investigates variables with potential to influence the tenure of those professionals at the time of hiring of the physician in the Primary Health Care Units. Brazil has experienced and unprecedented opening new medical schools, which lead an increase in offer of young physicians into the labour market. Although this gain in workforce, these professionals are initializing its medical career, and the majority will pass through medical residency programs, outside PHC services. Our model helps to understand the termination of the contracts in PHC as a phenomenon intrinsic the professional career of the physician, guiding public policies to study investments in specializing doctors with characteristics that increase its probability to stay on PHC and training young doctors to work in PHC settings.

We agree with reviewer about collinearity between variables ‘age at hire’ and ‘professional experience’. These variables were assessed in two distinct hierarchical levels, and previous study shows (Bourget et al, 2022 [11]) that collinearity did not affect the interpretability of the model. We also maintained these two variables in the same model because of its importance to understanding physician turnover considering the trajectory of the professional through the services.

As pointed by reviewer, we adjusted the terms and language in whole manuscript.

*2. Whole manuscript: My major concern – beside the fact that the results do not seem very relevant to me – is that several concepts are mixed in this manuscript. In the title, “turnover” in mentioned. The abstract mainly refers to “tenure”. Only in the conclusion, “turnover” is brought up again.*

*On the one hand, the outcome of this study is defined the “length of time from the date of hire to the termination of the physician’s employment in PHC services” (line 96). This sounds like “job tenure” (and a continuous variable) to me. On the other hand, in the results, protective factors against termination are identified, regardless of the duration, i.e., the job tenure. This sounds like “turnover” (and a dichotomous variable) to me.*

*Therefore, my suggestion is to adjust title, abstract, and the whole text (maybe even statistical analyses) completely to the concept of “job tenure” OR to the concept of “turnover” including a definition in the introduction and background.*

Our response: We modified 2^nd^ paragraph page 6 to explain the outcome of the study and the state variable of the survival analysis, as we can see:

“The outcome analyzed was the difference between the date of initial employment and the date of the physician’s termination of contract from PHC up until the date of the closing of the database in July 17st, 2020. The termination of contract is the situation of the physician’s employment contract within the institution. regardless of whether the termination was the choice of the professional or of the employer. This is the state variable of the survival analysis.”

*3. Introduction: Please extend the introduction and background. In this section, you might want to present prior evidence on factors that are associated with job tenure/retention/intention to stay OR turnover behaviour among health care professionals. Before this background, you might want to state more explicitly how your research contributes to the field.*

Our response: We presented the factors that are related and relevant to the tenure of primary care physicians in page 4, second paragraph (lines 77-82).

*4. Please state a concrete study aim in your manuscript.*

Our response: We corrected the aim in the manuscript regarding the objective presented in the abstract of the manuscript (page 4, 3^rd^ paragraph).

**Minor comments**

*5. Section “Variables, outcome definitions and ethical considerations”: In the abstract, you explain: “Human resource data from two distinct databases were used.”*

*a) As I could not find this information in the methods-section, please insert it there.*

*Our response: This statement was presented in Page 5, 1^st^ paragraph (lines 99-105).*

*b) Please describe who collected the data for the two databases, respectively.*

Our response: The database which came from employing organization is managed by the human resources sector, who was responsible to grant the data to the authors of the study after ethics approval. The second database, which came from Brazil Demography Study 2020 [20] are managed by National Commission of Medical Residency (CNRM), Brazilian Medical Association (AMB) and all the Medical Regional Councils (CRMs) (page 5, 1^st^ paragraph).

*c) Please give more specific information on the variables, e.g., gender: Was there a third option? What kinds of specialisations were given – or was it an open text field? How was professional experience operationalised?, How was workload operationalised?*

*d) Please specify if you did some recoding of the variables (e.g., dichotomisation) – and if so, how you did that.*

Our response (issues c) and d)): The variable “gender” was obtained from the regional database of the human resources sector of the employers, without a third option. For the calculation of the variable “professional experience”, we used the dates of graduation in medicine and the date of hiring of the professional in the PHC service, available in the national database of the study of the Medical Demography of Brazil 2020 [20] and the human resources sector of Social Organizations, respectively.

In the categorization of the “specialization” variable, extracted from the national database of the Medical Demography of Brazil 2020 study (15), physicians who obtained their title of specialist granted by specialty societies through the Brazilian Medical Association or by the Brazilian Medical Association were considered specialists. Completion of Medical Residency programs accredited by the National Commission for Medical Residency (CNRM). (19) Specialization in Family and Community Medicine, Gynecology and Obstetrics and Pediatrics were selected as categories apart from other specialists, given that its practice closely to PHC services in Brazil (13,15).

Dichotomizations of the variables ‘age at hire’, ‘professional experience’ and ‘salary’ were applied through the analysis of the distribution of the continuous variables and interquartile intervals.

*6. Page 5, lines 108-110: Please state the number of the Ethics Committee approval.*

Our response: We included all the number of the Ethics Committee approvals (Page 7, 3^rd^ paragraph, Lines 168-178)

*7. Page 5, line 114: Please give names of enterprises distributing the software you used.*

Our response: We included the names of enterprises of the statistical software used (page 6, 4^th^ paragraph, line 147)

*8. Page 6, line 140: Please explain the term “person-time”.*

*9. Page 6, line 141: Please revise number “26,113.741 months”.*

Our response (issues 8 and 9): We modify the term “person-time” (measurement that consider the number of people in the study and the amount of time each person spends in the study - time-at-risk) in the paragraph to indicate the incidence rate of termination the contract in Primary Health Care Units (page 8, 1^st^ paragraph, line 182-183).

*10. Page 6, lines 141f.: If the outcome of this study is the “length of time from the date of hire to the termination of the physician’s employment in PHC services”, how can participants still be “actively employed in PHC”? Please explain.*

Our response: We modify the paragraph indicating the outcome analyzed was the difference between the date of initial employment and the date of the physician’s termination of contract from PHC up until the date of the closing of the database in July 17st, 2020.

*11. Table 2: Please explain all abbreviations used in the table in a note below.*

Our response: We included all abbreviations used in the table (Page 27, line 598-00)

*12. Table 2: As far as I understand it, “workload” and “weekly working hours” are different concepts.*

*a) Please change the name of your variable or give a rationale why you operationalise “workload” as “weekly working hours”.*

*b) Please state if the weekly working hours are the hours specified in the contract or the actual working hours including overtime.*

Our response: We standardize the variable to ‘weekly workload’, which correspond the hours specified in the contract of the physician.

*13. In the title, you state that the data stem from a “representative cohort” – please explain how you ensured or checked that your sample is a representative one for all physicians in primary care in your study region.*

Our response: Using data (probability of observing the outcome in four years = 84,2% and HR adjusted in model VI) from reference [13] we estimate the power cox function in Stata Software (Significance level of 0.05 and power 0.8, two-sided test) which gave us a sample size of 1,387 individuals. Although we have removed this statement form the title, our study includes 65% from the Primary Health Care workforce, working in all different regions in Sao Paulo city, corresponding a 1,977 individual observed.

*14. Page 7, lines 149f.: Please name and define all your variables in the “Methods”-section.*

Our response: We corrected and adjusted all the variables naming in Methods section (Page 6, 1^st^ paragraph, line 124-129) and its definitions was presented in Table 1 (lines 128-129).

*15. You might want to start the discussion with a short summary of all your results.*

Our response: We remake the Results, excluding first paragraph (page 8), summarized demographic variables and professional experience in 2^nd^ paragraph (lines 189-193) and aspects of medical education program (page 9, 1^st^ paragraph, lines 199-203).

*16. Page 10, line 243: You may want to revise the beginning of the sentence.*

Our response: We corrected the sentence and the beginning the paragraph (page 12, 1^st^ paragraph), as follows:

In Brazil, the number in first-year medical students increased from 20,522 in 2013 to 37,346 in 2019, with 84% due to private schools, alerting to its capacity to train physicians who can work in PHC [19].

*17. Page 13, lines 308f.: “For instance, because the excluded physicians were hired by organizations other than the three employers studied …” I did not quite catch that fact before. Please explain in your methods why you focused on three employers only.*

Our response: We add this information in a new paragraph (page 5, 3^rd^ paragraph, lines 115-121). We selected theses three employers considering the percentual of the primary care workforce employed and because they allowed access to the anonymous information of physicians to carry out the study.
