# Supplementary material for: Effects of individual and organizational factors on job tenure of primary care physicians: A multilevel analysis from Brazil
Source: PLoS One. 2023 Apr 27;18(4):e0271655. doi: 10.1371/journal.pone.0271655 (PMC10138250; doi:10.1371/journal.pone.0271655)
Supplement: Attachment — Submitted filename: Response to Reviewers.docx [file pone.0271655.s003.docx]

March 10, 2023

**Revision of manuscript PONE-D-22-18937R1**

**Effects of individual and organizational factors on turnover of primary care physicians: A multilevel analysis from Brazil**

Dear Prof. André Ricardo Ribas Freitas

Academic Editor, Plos One

Thank you for the opportunity to submit a new revision of our manuscript to the Plos One, and for the insightful comments received.

This document includes all of our responses to reviewers' comments.

Please do not hesitate to contact me if you have further comments or questions.

Sincerely,

**Ivan Wilson Hossni Dias**

***Corresponding author***

**Reviewer #3**

Effects of individual and organizational factors on turnover of primary care physicians: A multilevel multivariate analysis from Brazil

**Purpose of the study**

This study aims to investigate the job tenure among physicians in primary care (n = 2,335). Organisational as well as individual factors are shown to be associated with job tenure.
The manuscript has improved. However, some of the comments were poorly addressed. Still, there are several issues to be optimised. Line numbers refer to the manuscript without tracked changes.

Our response: We thank the reviewer’s comments and contributions to improve the quality of the manuscript and we hope to address the issues pointed in this review. Line numbers of our responses refer to the manuscript with tracked changes.

**Major comments**

1. In lines 131f., you state: “The outcome analyzed was the difference between the date of initial employment and the date of the physician’s termination …”, and in the abstract, you speak of “job tenure”. In the results section, you name your outcome “termination” (e.g., lines 222, 228 and so on). Please – again – use your terms consistently after having defined them. Please consider to add the term “job tenure” in line 131 where you define your outcome (without giving it a proper name), e.g.:

“The outcome analyzed was “job tenure”, defined as the difference between the date of initial employment and the date of the physician’s termination …”

2) Connected to issue #1: If you analyze “job tenure”, you should name it in the title instead of “turnover”. It is a different concept. So, please change the title, e.g.:
Effects of individual and organizational factors on job tenure of primary care physicians: A multilevel multivariate analysis from Brazil

Our response: We have now modified the title, changing the term “turnover” to “job tenure” and we adjusted the definition of the outcome, including the term “job tenure” as suggested (page 6, line 136). We also changed lines 140 and 141 to improve the understanding of the event of interest of the survival analysis performed (time-to-event analysis), which is the termination of contract from a PHC service. We changed the term in line 229 and adjusted the first paragraph (lines 229-231), also adjusted to “job tenure” in line 237 (page 10); line 255 (page 11) and line 309 (page 13).

**Minor comments**

3. Line 39: Please add full stop.

Our response: We modify adding full stop (line 39, page 2).

4. Lines 93ff.: The sentence sounds strange. Please consider rephrasing it:
“The purpose of this study is to investigate the contextual and individual factors (…)”

Our response: We accept the suggestion and modified the sentence (line 93-94).

5. Tables 1 and 2 and 3 (!):

a) Please correct category definitions for age; the age of 60 comes up twice:

2- 30–60 -> should be 30– < 60 (?)

3- ≥ 60

b) The same problem occurs with professional experience:

1- 1–2 years -> should be 1– < 3 (?)

What about 2,5 years? Or were only full years stated without decimals? How were the numbers

rounded? Please explain in the manuscript.

2- 3–5 -> should be 3– < 5 (?)

3- 5–10 -> should be 5– < 10 (?)

4- More than 10 -> should be ≥ 10 (consistency)

c) The same problem occurs with professional experience. Please correct and use mathematical operators (e.g., instead of “more than”).

Our response: We changed the mathematical operators of the variables “age” and “professional experience” in all tables. As presented in table 1, the variable “professional experience” was calculated by the difference in years between the date of graduation and the date of initial employment (hiring) in PHC, and in our study it was considered as a continuous variable, allowing decimals in the intervals presented. We also explain the dichotomization process in page 6, lines 129-131.

6. Table 1 (and WHOLE MANUSCRIPT): Please speak of “weekly working hours” instead of “workload”, because it is a different concept. “Workload” means the amount of work to be done within a particular period of time. But you only refer to the time, not the amount of work (e.g., operationalised as extra hours/overtime). So please change the wording or quote a prior definition that fits to your use of “workload”.

Our response: We added a quote a prior adopted in the study to the concept of “workload” (lines 127-129, page 6, first paragraph) as follows:

“In this study, the variable “workload” refers to the weekly working hours stablished in the contract between the PHC physician and the employer organization.”

7. You explained in your responses: “Dichotomizations of the variables ‘age at hire’, ‘professional experience’ and ‘salary’ were applied through the analysis of the distribution of the continuous variables and interquartile intervals.” Please give this information in the manuscript too.

Our response: We have now changed the paragraph that describes the variables of the study and included the information about the dichotomization process of the variables “age at hire”, “professional experience” and “salary” (page 6, lines 129-131).

8. Line 165: “managed”?

Our response: We changed the term to “handled” (line 171, page 7).

9. Line 177: First mentioning of “SPDM and ASF”? Please explain abbreviations in the text.

Our response: We added an explanation in lines 183-184 (page 8). As presented in table 1, SPDM (Associação Paulista para o Desenvolvimento da Medicina) and ASF (Associação Saúde da Família) are names of the employers organizations of health workforce in Sao Paulo capital.

10. Table 2 (additional to issues mentioned above):

a) Please use n (lower case) instead of N to describe your sample.

b) Please add commas between definition and next abbreviation, as you did in Table 1.

Our response: We changed the description of the sample to lower case (Table 2) and we added the commas between definition and next abbreviation (line 601 Table 2, page 28)

11. Line 201: Please do not begin a sentence with a number, e.g., “A total of 418 …”

Our response: We modify the sentence as appointed (line 209, page 9)

12. Table 3:

a) Please write “analysis” with a lower-case a in the Table title.

b) Please use n (lower case) instead of N to describe your sample.

c) Please write “95% CI” instead of “CI 95”.

d) AGAIN, please explain all abbreviations in a footnote (PHC, CI).

Our response: We changed Table 3’s title to correct the term “analysis” also presented in line 586 of tables titles descriptions. We changed to lower case de description of the sample (line 603, Table 3). We rewrite the Confidence Interval and add the explanations of the abbreviation in a footnote of the table (line 604).

13. Line 255: Please delete comma.

Our response: We modified the sentence and eliminated the comma (page 11, line 261)

14. Conclusions: As far as I know, you do not need to explain your abbreviations again in the conclusions, so please delete the explanations and use the abbreviations.

Our response: We deleted the explanations (lines 368-371).

15. Line 362: Please delete “representative”, as you have not referred to this characteristic of your sample in the manuscript before.

Our response: We deleted the term from Conclusion (lines 372, page 16).

16. Please complete your list of abbreviations.

Our response: We changed the list of abbreviations adding the employing organization names (ASF Associação Saúda da Família and SPDM: Associação Paulista para o Desenvolvimento da Medicina); CI (Confidence Interval) and the following sources abbreviations (BMD: Brazilian Medical Demography and HRD Human Resources Database)
